# Associations between nutritional factors and *KRAS* mutations in colorectal cancer: a systematic review

**DOI:** 10.1186/s12885-020-07189-2

**Published:** 2020-07-28

**Authors:** Achraf El Asri, Btissame Zarrouq, Khaoula El Kinany, Laila Bouguenouch, Karim Ouldim, Karima El Rhazi

**Affiliations:** 1 Laboratory of Epidemiology and Research in Health Sciences, Faculty of Medicine and Pharmacy, Sidi Mohammed Ben Abdallah University, Fez, Morocco; 2 Medical Genetics and Oncogenetics Unit, Hassan II University Hospital, Fez, Morocco; 3 Teacher’s Training College (Ecole Normale Superieure), Department of Biology and Geology, Sidi Mohammed Ben Abdallah University, Fez, Morocco; 4 Cancer Research Institute, Fez, Morocco

**Keywords:** *KRAS* mutations, Colorectal cancer, Diet, Nutrients, Foods

## Abstract

**Background:**

Between 30 and 50% of colon tumors have mutations in the Kirsten-*ras* (*KRAS*) gene, which have a large nutritional attributable risk. Despite its high frequency in colorectal cancer (CRC), data to support specific associations between *KRAS* mutations in CRC and diet are sparse. Here, we conducted a systematic review to summarize the current epidemiological evidence on the association between various dietary factors and *KRAS* mutations.

**Methods:**

PubMed, Science Direct, and Cochrane databases were searched for relevant studies published until December 31, 2019, using inclusion and exclusion criteria in accordance with PRISMA guidelines. We analyzed the studies to find associations between nutritional factors and CRC tumors with *KRAS* mutations in humans.

**Results:**

We identified 28 relevant studies to include in this systematic review. In-depth analyses showed unclear associations between nutritional factors and *KRAS* mutations in CRC. Most epidemiological studies in the same nutrient or food often reported conflicting and/or inconclusive findings, whereas for some dietary factors, the results were homogeneous.

**Conclusions:**

Further research using a more robust prospective cohort study is needed to lend more credence to the epidemiological associations found between *KRAS* mutations and dietary factors.

## Background

Colorectal cancer (CRC), which usually presents as colorectal adenocarcinoma, is the third most commonly diagnosed cancer and the second most deadly cancer worldwide [1]. Both mutations and epigenetic modifications in oncogenes and tumor suppressor genes lead to the development of cancer [2, 3]. In CRC, the key genes include *TP53 (*tumor protein 53), *APC (*adenomatous polyposis coli)*,* and *KRAS* (Kristen rat sarcoma) [4].

Of the key genes, *KRAS* mutations are the most widely known, as they are mainly localized in codons 12 and 13, which were among the first linked to the pathogenesis of colon cancer, and have been found in about 42,6% of CRC cases worldwide [5–7].

Because *KRAS* mutations are recognized as an early event in colorectal carcinogenesis, and are associated with a worse prognosis and resistance to cetuximab therapy [5, 8], they may be helpful in screening and early diagnosis of CRC [9]. Furthermore, *KRAS* mutations play an important role in targeted therapy response [10]. Clinical trials have revealed that patients with wild-type *KRAS* (*KRAS*^−^) had better clinical response in terms of prolonged median progression-free survival and overall response rates compared with those with mutant *KRAS* (*KRAS*^+^) [11, 12].

Despite the frequency of *KRAS* mutations in CRC, data on their etiology are sparse, and their occurrence and persistence have been blamed on many risk factors. Although heredity may play a role, a history of exposure to environmental risk factors, including dietary factors, has also been suggested [13]. In fact, there is an interaction of cell molecular changes and environmental factors, with a great contribution of diet components [14]. Therefore, epidemiological studies have been conducted to study possible relationships between known or suspected nutritional factors related to the risk of CRC and the occurrence and persistence of *KRAS* mutations. Biologically plausible mechanistic studies in vitro models [15, 16], or in animal models of CRC [17, 18] have also been conducted to understand how nutritional factors may influence the risk of mutation.

Here, we conducted a systematic review to summarize the current epidemiological evidence on the relationship between various dietary factors and *KRAS* mutations on human populations. Understanding how *KRAS* mutations arise in colorectal tumors may provide valuable clues for prevention strategies.

## Methods

### Search strategy

The search was conducted in accordance with the Preferred Reporting Items for Systematic Reviews and Meta-Analyses (PRISMA) guidelines for systematic reviews [19] to identify studies reporting associations between nutritional factors and *KRAS* mutations in CRC worldwide.

We conducted an exhaustive search for English literature studies in the PubMed (https://www.ncbi.nlm.nih.gov/), Cochrane (www.thecochranelibrary.com), and ScienceDirect (https://www.sciencedirect.com) databases. The main search terms included “nutritional factors” or “nutrition” or “nutrient” or “diet” or “aliment” or “food” AND “KRAS mutation” or “Kirsten rat sarcoma” or “K-ras” or “Ki-ras” or “KRAS2” or“K-ras2” or “Ki-ras2” AND “colorectal cancer” or “colorectal carcinoma” or “colon” or “rectum.” To avoid missing any articles, the search was cast more widely, with references of included articles also individually checked. All identified studies published until December 31, 2019 were considered.

### Inclusion and exclusion criteria

Studies were included if they explored the association between nutritional factors and CRC tumor with *KRAS* mutations in human subjects. We excluded experimental studies on human or animal cells and studies of *KRAS* mutations in other types of cancer. Only observational studies were included (case series, case-control, and cohort studies).

### Data extraction

All identified studies were independently reviewed by two authors for relevance of the inclusion/exclusion criteria. The two authors extracted specific data from each study, including the name of the first author, country, study design, number of participants, year of publication, exposure and confounding factors, specific characteristics and outcomes, main findings, and effects.

### Quality assessment

The quality of the included studies was assessed using PRISMA guidelines [19]. Study quality was assessed according the following criteria: accuracy and validity of the questions (answers per evidence) and the representability of the studied population. Study quality was also assessed according to the strength of the findings in relation to type of study design (level) and the study’s methodological weaknesses (the biases and limitations of each study) [20].

## Results

**Fig. 1:**
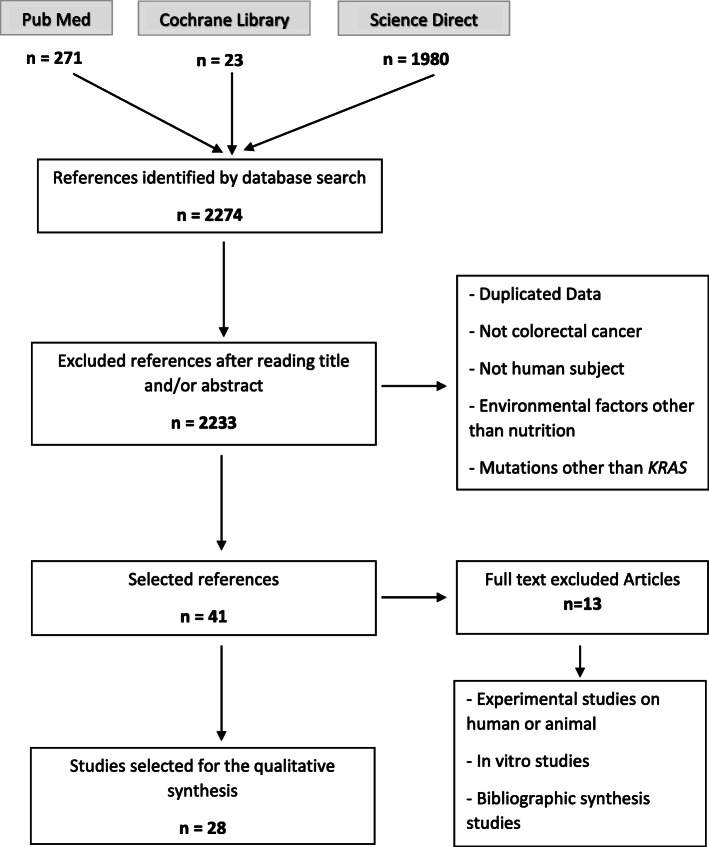
Flow diagram of process of systematic literature search in accordance with PRISMA guidelines

The literature search identified 2274 studies. After exclusion of duplicate studies from PubMed, Cochrane, and ScienceDirect searches, and after stepwise exclusion of research outside the scope of our review (mostly laboratory and animal studies, research involving other cancers, and studies focused on cancer treatment or survival), only 41 studies remained for further in-depth analysis through reading of the full text. Thirteen articles were excluded because they were experimental studies on human or animal, or in vitro studies, or bibliographic synthesis studies. This resulted in 28 original studies published between 1997 and 2019 for inclusion in our systematic review. The PRISMA diagram for the systematic review process is shown in Fig. 1.

The quality assessment of selected studies is presented in Table 1. The included studies in the current systematic review have an acceptable quality assessment. Nearly two thirds of them were large cohort or large case control studies.

**Table 1:** Quality assessment of published papers on nutritional factors and *KRAS* mutations in colorectal cancer worldwide

Author, Year, and Reference Number	Relevant to This Review	Aims Clearly Stated	Appropriate Study Method	Sample Representative of Target Population	Confounding and Bias Considered	Good Response Rate	Were Questions Piloted	Were Tables and Figures Understandable	Can Results Be Applied to Local Situation?	Accepted as Type IV Evidence
He et al., 2019 [21]	Yes	Yes	Yes	Yes	Yes	Yes	Yes	Yes	Yes	No (Type III)
Keum et al., 2019 [22]	Yes	Yes	Yes	Yes	Yes	Yes	Yes	Yes	Yes	No (Type III)
Mehta et al., 2017 [23]	Yes	Yes	Yes	Yes	Yes	Yes	Yes	Yes	Yes	No (Type III)
Carr et al., 2017 [24]	Yes	Yes	Yes	Yes	Yes	Yes	Yes	Yes	Yes	No (Type III)
Hogervorst et al., 2014 [25]	Yes	Yes	Yes	Yes	Yes	Yes	Yes	Yes	Yes	No (Type III)
Jung et al., 2014 [26]	Yes	Yes	Yes	No	Yes	Yes	Yes	Yes	Yes	No (Type III)
Gilsing et al., 2013 [27]	Yes	Yes	Yes	Yes	Yes	Yes	Yes	Yes	Yes	No (Type III)
Kamal et al., 2012 [28]	Yes	Yes	Yes	No	No	Yes	No	Yes	Yes	No (Type III)
Razzak et al., 2012 [29]	Yes	Yes	Yes	Yes	Yes	Yes	Yes	Yes	Yes	No (Type III)
Ottini et al., 2011 [30]	No	Yes	Yes	No	–	–	–	–	No	No (Type V)
Naguib et al., 2010 [31]	Yes	Yes	Yes	No	No	Yes	Yes	Yes	No	No (Type V)
Slattery et al., 2010 [32]	Yes	Yes	Yes	No	Yes	No	Yes	Yes	Yes	No (Type III)
Schernhammer et al., 2008 [33]	Yes	Yes	Yes	Yes	Yes	Yes	Yes	Yes	Yes	No (Type III)
Weijenberg et al., 2007 [34]	Yes	Yes	Yes	Yes	Yes	Yes	Yes	Yes	Yes	No (Type III)
Bongaerts et al., 2006 [35]	Yes	Yes	Yes	Yes	Yes	Yes	Yes	Yes	Yes	No (Type III)
Wark et al., 2006 [36]	Yes	Yes	Yes	No	Yes	Yes	Yes	Yes	Yes	No (Type III)
Brink et al., 2005 [37]	Yes	Yes	Yes	Yes	Yes	Yes	Yes	Yes	Yes	No (Type III)
Brink et al., 2005 [38]	Yes	Yes	Yes	Yes	Yes	Yes	Yes	Yes	Yes	No (Type III)
Brink et al., 2004 [39]	Yes	Yes	Yes	Yes	Yes	Yes	Yes	Yes	Yes	No (Type III)
Howsam et al., 2004 [40]	Yes	Yes	Yes	No	Yes	Yes	Yes	Yes	Yes	No (Type III)
Laso et al., 2004 [41]	Yes	Yes	Yes	No	No	Yes	Yes	Yes	Yes	No (Type III)
Slattery et al., 2002 [42]	Yes	Yes	Yes	Yes	Yes	Yes	Yes	Yes	Yes	No (Type III)
Slattery et al., 2001 [43]	Yes	Yes	Yes	Yes	Yes	Yes	Yes	Yes	Yes	No (Type III)
Slattery et al., 2000 [44]	Yes	Yes	Yes	Yes	Yes	Yes	Yes	Yes	Yes	No (Type III)
Kampman et al., 2000 [45]	Yes	Yes	Yes	No	Yes	Yes	Yes	Yes	Yes	No (Type III)
O’Brien et al., 2000 [46]	Yes	Yes	Yes	No	No	Yes	Yes	Yes	Yes	No (Type V)
Martinez et al., 1999 [47]	Yes	Yes	Yes	No	Yes	Yes	Yes	Yes	No	No (Type V)
Bautista et al., 1997 [48]	Yes	Yes	Yes	No	Yes	Yes	Yes	Yes	Yes	No (Type III)

Among 28 articles included for review, 12 studies were case cohort studies, 12 were case-control studies, 3 were case series studies, and 1 was a case report study. All studies had an objective of determining a link between known or suspected nutritional factors for CRC and *KRAS* mutations. Some articles investigated a single type of diet, whereas some tackled multiple ones. Investigated dietary factors included meat, fruits and vegetables, fiber, dairy products, coffee and tea, acrylamide foods, alcoholic beverages, and organochlorine compounds, as well as numerous nutrients, including vitamins (A, B1, B2, B6, B9, B12, D, E), calcium, animal protein, heme iron, and fat. Table 2 summarizes the main findings, and the sections below summarize the most relevant findings for each of the foods and nutrients studied (arranged in alphabetical order).

**Table 2:** Main results of included studies

Author, Year, and Reference	Study Design	Country and Setting	Sample size	Ethnicity	Main Focus	Relevant Exposures	Confounder Factors	Comparison Groups	Main Findings and Effects
He et al., 2019 [21]	Two large prospective cohort studies: Nurses’ Health Study (NHS), 1980–2012) and Health Professionals Follow-up Study (HPFS), (1986–2012).	USA: - Nurses’ Health Study (NHS): 11 US States (California, Connecticut, Florida, Maryland, Massachusetts, Michigan, New Jersey, New York, Ohio, Pennsylvania, and Texas). - Health Professionals Follow-up Study (HPFS): 50 US states.	- A total of 138,793 participants were included: 90,869 from the NHS and 47,924 from the HPFS. - 1337 cases with data for KRAS Mutation.	Unspecified	Dietary intake of fiber, whole grains and risk of colorectal cancer.	- Total fiber (per 5 g/day) - Cereal fiber (per 5 g/day) - Fruit fiber (per 5 g/day) - Vegetable fiber (per 5 g/day) - Whole grain (per 20 g/day).	Age, family history of CRC, history of lower gastrointestinal endoscopy, smoking, body mass index, physical activity, alcohol intake, regular aspirin use, regular multivitamin use, total folate intake, calcium intake, vitamin D intake, glycemic load, processed red meat intake, hormone use.	- Group I: KRAS+ - Group II: KRAS-	No association
Keum et al.,2019 [22]	Two ongoing prospective cohort studies: the Nurses’ Health Study (NHS) (1980–2010), and the Health Professionals Follow-up Study (HPFS) (1986–2010).	USA: - NHS: 11 US States. - HPFS: 50 US states.	- 88,506 women and 47,733 men. - 853 colon cancer cases.	Unspecified	Calcium intake and colon cancer risk subtypes by tumor molecular characteristics.	Total calcium intake (mg/day).	Age, questionnaire cycle, sex; Caucasian (yes vs. no), family history of colorectal cancer, history of sigmoidoscopy/colonoscopy, regular aspirin use, smoking, BMI, physical activity, 25-hydroxyvitamin D scores, intakes of energy, alcohol, red and processed meat and folate.	- Group I: KRAS+ - Group II: KRAS-	No association
Mehta et al., 2017 [23]	Two ongoing cohorts, the Health Professionals Follow-up Study (HPFS) and the Nurses’ Health Study (NHS).	USA: - NHS: 11 US States. - HPFS: 50 US states.	- 137,217 participants (47,449 men and 89,768 women). - 1285 tumors for KRAS mutation status.	Unspecified	Western and prudent dietary patterns and risk of CRC.	Western and prudent dietary patterns score.	Age, sex, CRC family history, history of previous lower gastrointestinal endoscopy, smoking, body mass index, physical activity, NSAID, and total caloric intake.	- Group I: KRAS+ - Group II: KRAS-	No association with tumors harboring KRAS mutation.
Carr et al., 2017 [24]	Case–control study	Southwest of Germany	- 2449 cases and 2479 controls.	Unspecified	Associations of red and processed meat intake with major molecular pathological .	Red and processed meat (frequency: times/day).	Age, sex, school education, BMI, family history of colorectal cancer, history of large–bowel endoscopy, participation in health checkup, smoking, ever regular use of NSAIDs, fruit intake, and wholegrain intake.	- Group I: KRAS+ - Group II: KRAS-	Positive association with higher red and processed meat intake and KRAS mutation (OR > 1 time/day vs ≤ 1 time/week: 1.49, 95% CI 1.09–2.03).
Hogervorst et al., 2014 [25]	Case cohort study embedded in the Netherlands Cohort Study on diet and cancer (NLCS).	Netherlands (204 municipalities with computerized population registries).	- 120,852 participants (58,279 men + 62,573 women) - Subcohort (*n* = 5000) - 733 CRC cases were available for the molecular analysis.	Unspecified	Acrylamide and CRC risk characterized by mutations in *KRAS* and *APC.*	- Acrylamide intake (g/day).	Age, smoking, BMI, family history of CRC, total energy intake.	- Group I: *KRAS+* - Group II: *KRAS*^*−*^	- Positive association with acrylamide intake among men (HR [4th quartile vs. 1st] = 2.12; 95% CI, 1.16–3.87; *p* = .01).
Jung et al., 2014 [26]	Two cohorts, the Health Professionals Follow-up Study (HPFS) and the Nurses’ Health Study (NHS) (1986–2008).	USA: - NHS: 11 US States. - HPFS: 50 US states.	- 140,418 participants. - 1059 incident CRC cases with tumor molecular data.	Unspecified	Association between vitamin D and CRC risk.	- Predicted vitamin D score (ng/mL)	Age, sex, family, history of endoscopy, aspirin use, smoking, intake of total fruits and vegetables, total calories.	- Group I: *KRAS*^+^ - Group II: *KRAS*^−^.	Negative association between higher predicted vitamin D score and *KRAS* mutation (HR = 0.70; 95% CI, 0.50–0.98).
Gilsing et al., 2013 [27]	Cohort Study initiated in September 1986.	Netherlands (204 municipalities with computerized population registries).	- Case subjects were enumerated from the entire cohort (120,852 men and women). - the accumulated person years of the entire cohort were estimated from a random subcohort of 5000 men and women. - 733 CRC cases were available for the molecular analysis.	Unspecified	Dietary heme iron intake and risk of CRC with mutations in *APC* and *KRAS* and *p53* overexpression	- Heme iron intake (g/day)	Age, sex, BMI, family history of CRC, smoking, nonoccupational physical activity, total energy intake, alcohol consumption, total vegetable consumption	- Group I: wild-type *KRAS* - Group II: activating mutant *KRAS*	Positive association with heme iron intake (HR = 1.71; 95% CI, 1.15–2.57; *P* = .03)
Kamal et al., 2012 [28]	Retrospective cohort study	Egypt. Kasr El Aini Hospital, Cairo University.	80 CRC patients (56 males and 24 females).	Unspecified	Associations between *KRAS* mutation and potential variables known or suspected to be related to the risk of CRC.	- Meat, green leafy vegetables, tea, and coffee at < 3 times/week versus more than 3 times/week. - Red blood cell folic acid (ng/mL).	not mentioned	- Group I: *KRAS+* - Group II: *KRAS*^*−*^	Potential link between folic acid and *KRAS* mutation, suggesting that folic acid may be a risk factor for *KRAS* mutation development - OR for folic acid was 0.983 for each 1 ng/mL higher folate.
Razzak et al., 2012 [29]	Cohort study from the Iowa Women’s Health Study.	Iowa, USA.	- *n* = 41,836 - 514 incident CRC cases were available for the molecular marker assays.	Caucasian women.	Associations between dietary folate, vitamin B6, vitamin B12, and methionine with different pathways in CRC.	- Folate (μg/day) - Vitamin B6 (mg/day) - Vitamin B12 (μg/day) - Methionine (g/day).	Age,, BMI, waist-to-hip ratio, smoking status, exogenous estrogen use, physical activity level, and daily intake of total energy, total fat, sucrose, red meat, calcium, methionine, vitamin E, alcohol.	- Group I: *KRAS+* - Group II: *KRAS*^*−*^	None of the dietary exposures were associated with *KRAS*-defined CRC subtypes.
Ottini et al., 2011 [30]	Case study	Italy	1 individual (King Ferrante I of Aragon).	Caucasian	Explanation of the death of King Ferrante I	Carbon (δ-^13^C) and nitrogen (δ-^15^N) isotope analysis.	not mentioned	–	Possible abundance of dietary carcinogens, related to meat consumption, could explain *KRAS* mutation causing the colorectal tumor that killed Ferrante I more than 5 centuries ago.
Naguib et al., 2010 [31]	Case series.	Norfolk, United Kingdom.	−25,639 from The EPIC Norfolk cohort (1993–1997). - 202 CRC cases were tested for Kras mutations.	Unspecified	Associations between *BRAF* and *KRAS* mutations and clinicopathologic, lifestyle, and dietary factors in CRC.	- Alcohol (g/day) - Meat (g/day), including red meat, red processed meat, white meat, white fish, fatty fish - Fruit and vegetables - Fat, total fat, PUFA, MUFA, SFA -Vitamins B2, B3, B6, B9, B12, C, and D - Fiber and macronutrients: total energy (MJ/day), carbohydrates (g/day), protein (g/day), nonstarch polysaccharide (g/day), calcium (mg/day).	Not mentioned	- Group I: Patients with *KRAS*^*+*^ - Group II: Patients with *KRAS*^*−*^	- *KRAS* mutation was associated with increased white meat consumption (*P* < .001; ANOVA) - *KRAS* (G to A) associations were found in individuals with significantly lower consumption of fruits or vegetables (*P* = .02).
Slattery et al., 2010 [32]	Case control study of participants in Kaiser Permanente Medical Care Program study	Northern California and Utah, USA.	- 951 cases - 1205 controls	82% white, non-Hispanic, 4.1% African American, 7.6% Hispanic, 4.6% Asian, 0.7% American Indian, and 1% multiple races/ethnicity.	Diet, physical activity, and body size associations with rectal tumor mutations and epigenetic changes.	- Foods and dietary patterns involving dairy high fat, low fat, fruit, vegetables, red meat, fish, whole grains, refined grains, Western diet, prudent diet. - Nutrients: calories, PUFA, MUFA, SFA, trans fats, omega-3 fats, animal protein, vegetable protein, carbohydrates, dietary fiber.	Age, sex, recent aspirin use, long-term activity level, pack-years of cigarette smoking, dietary calcium, energy intake.	- Group I: CpG Island methylator phenotype CIMP+ - Group II: TP53 mutation - Group III: *KRAS2*^*+*^ mutations - Group IV: controls.	- High levels of vegetable intake reduced risk of KRAS mutations (OR = 0.60; 95% CI, 0.40–0.89; *P* < .01) - Dietary fiber was associated with reduced risk of KRAS rectal tumor mutations - Prudent dietary pattern significantly reduced the *KRAS* mutation risk (OR = 0.68, 95% CI, 0.47–0.98; *P* < .03). - No significant result for the other factors.
Schernhammer et al., 2008 [33]	Two prospective cohort studies: NHS and HPFS.	USA: - NHS: 11 US States. - HPFS: 50 US states.	- 88,691 women and 47,371 men. - 669 incident cases of CRC were available for the molecular analysis.	Unspecified	Association between dietary folate intake, vitamin B, and incidence of *KRAS* mutation in colon cancers.	- Folate (μg/day).	Age, sex, energy intake, screening sigmoidoscopy, family history, aspirin use, smoking, physical activity, BMI in 5 categories, colon polyps, beef intake, calcium intake, multivitamin use, alcohol use, and intake of vitamin B6, B12, and methionine.	- Group I: *KRAS*^−^ cancer cases - Group II: *KRAS*^+^ cancer cases	Low folate and vitamin B6 intakes were associated an increased risk of colon cancer, but these effects did not differ significantly by *KRAS* mutational status.
Weijenberg et al., 2007 [34]	Cohort study: Netherlands Cohort Study on diet and cancer (NLCS).	Netherlands (204 municipalities with computerized population registries).	- 531 incident cases of CRC were available for the molecular analysis.	Unspecified	Baseline fat intake versus risk of colon and rectal tumors with some gene alterations.	- Fat variables (g/day), including total fat, SFA, MUFA, PUFA, linolenic acid, linoleic acid.	Age, sex, BMI, family history of CRC, daily energy intake, daily linoleic acid intake, daily calcium intake, smoking.	- Group I: colon cancer with no gene aberrations - Group II: colon cancer with activating *KRAS* gene mutations.	- No association with total, saturated, MUFA, and PUFA - Linoleic acid showed a positive association with *KRAS* mutation (RR = 1.41; 95% CI, 1.18–1.69).
Bongaerts et al., 2006 [35]	Prospective Cohort study: Netherlands Cohort Study on diet and cancer (NLCS).	Netherlands (204 municipalities with computerized population registries).	- The cohort included 58,279 men and 62,573 women. - 578 incident cases of CRC were available for the molecular analysis.	Unspecified	Associations between consumption of alcohol and alcoholic beverages and risk of CRC without and with specific *KRAS* gene mutations.	- Alcohol consumption: total alcohol (g/day), beer (glasses/week), wine (glasses/week), liquor (glasses/week).	Age, family history of CRC, BMI, calcium intake, linoleic intake, smoking, total alcohol consumption.	- Group I: colon cancer, *KRAS*^*+*^ - Group II: colon cancer, *KRAS*^−^ - Group III: rectal cancer, *KRAS*^+^ - Group IV: rectal cancer, *KRAS*- - Men and women analyzed separately.	- No association between alcohol and *KRAS* mutations - Positive association with beer drinking (RR: 3.48; 95% CI, 1.1–11.0).
Wark et al., 2006 [36]	Case-control study	Netherlands (outpatient clinics of 10 hospitals).	- 658 cases - 709 controls	Unspecified	Associations between diet, lifestyle, and *KRAS* mutations.	- Foods (g/day): dairy products, red meat, tea - Macronutrients (g/day): total dietary fat, PUFA, MUFA, protein - Micronutrients (mg/day): calcium, vitamin B2.	Sex, age, total energy.	- Group I: patients with *KRAS*^+^ - Group II: patients with *KRAS*^−^ - Group III: controls.	No significant results: - Red meat OR = 1.70 (95% CI, 0.94–3.09), potential risk, not statistically significant result - Total dietary fat OR = 0.55 (95% CI, 0.28–1.06) - PUFA OR = 0.58 (95% CI, 0.31–1.10) - No differences versus risk of KRAS adenomas could be detected for other factors.
Brink et al., 2005 [37]	Cohort study: Netherlands Cohort Study on diet and cancer (NLCS).	Netherlands (204 Dutch municipalities with computerised population registries).	− 2948 subcohort members. − 608 incident colon and rectal cancer cases were available for the molecular analysis.	Unspecified	Association between meat and *KRAS* mutations in sporadic colon and rectal cancer.	Meat (g/day): total fresh meat, beef, pork, minced meat, liver, chicken, other meat, meat product, fish.	Age, sex, Quetelet Index, smoking, energy intake, family history of CRC.	- Group I: patients with *KRAS* mutation - Group II: patients with G > C or G > T activating *KRAS* mutation - Group III: patients with G > A activating *KRAS* mutation - Group IV: patients with *KRAS*^*−*^.	- For meat products, positive association shown (RR for highest vs lowest quartile of intake = 2.37; 95% CI, 0.75–7.51; *P* = 0.07) - No clear associations were observed for total fresh meat, different types of fresh meat, meat products, and fish.
Brink et al., 2005 [38]	Cohort study: Netherlands Cohort Study on diet and cancer (NLCS).	Netherlands (204 municipalities with computerized population registries).	- 3048 Subcohort members (1475 men and 1573 women). - 608 incident CRC cases were available for the molecular analysis.	Unspecified	Association between dietary folate and specific *KRAS* mutations in CRC.	- Folate (μg /day).	Age, sex, BMI, smoking, alcohol, fresh meat, energy intake, family history of CRC, vitamin C, iron, fiber.	- Group I: colon cancer, *KRAS*^+^ - Group II: colon cancer, *KRAS*^−^ - Group III: rectal cancer, *KRAS*^+^ - Group IV: rectal cancer, *KRAS*^−^.	- For women: folate intake was associated with an increased risk of *KRAS* mutation G > T and G > C (RR = 2.69; 95% CI, 1.43–5.09) but inversely associated with G > A (RR = 0.08; 95% CI, 0.01–0.53) - For men: folate intake was associated with decreased risk of *KRAS* mutation (RR = 0.40; 95% CI, 0.17–0.89).
Brink et al., 2004 [39]	Cohort study: Netherlands Cohort Study on diet and cancer (NLCS).	Netherlands (204 municipalities with computerized population registries).	- 2948 Subcohort members. - 608 incident CRC cases were available for the molecular analysis.	Unspecified	Associations between dietary intake of various fats and specific *KRAS* mutations in CRC.	Fat variables (g/day): - Total fat - SFA - MUFA - PUFA - Linolenic acid - Linoleic acid.	Age, sex, Quetelet Index, smoking, energy intake, family history of CRC.	- Group I: colon cancer, *KRAS*^+^ - Group II: colon cancer, *KRAS*^−^ - Group III: rectal cancer, *KRAS*^+^ - Group IV: rectal cancer, *KRAS*^−^.	- No association with intake of total fats, SFA, and MUFA - Positive association with high intake of PUFA and linoleic acid (RRs for 1 SD of increase of PUFA and linoleic acid = 1.21; 95% CI, 1.05–1.41; and 1.22; 95% CI, 1.05–1.42).
Howsam et al., 2004 [40]	Case control study	Barcelona, Catalonia, Spain.	Subsample of cases (*n* = 132) and hospital controls (*n* = 76) selected from a larger case-control study.	Unspecified	Association between risk of CRC and exposure to organochlorine compounds.	Different types of organochlorines: - p,p’-DDE (low, medium, high) - α-HCH (low, medium, high) - PCB-28 (low, medium, high) - PCB-118 (low, medium, high).	Age, sex, BMI, energy intake.	- Group I: *KRAS*^−^ - Group II: *KRAS*^+^	- Exposure to mono-ortho PCB-28 and PCB-118 increased risk of tumor *KRAS*^+^ - PCB-28 OR = 2.83 (95% CI, 1.13–7.06). - PCB-118 OR = 1.64 (95% CI, 0.67–4.01).
Laso et al., 2004 [41]	Case-control study	Catalonia, Spain.	- 246 cases - 296 controls	Unspecified	Association between specific micronutrient intake and CRC and *KRAS* mutation	- Fiber (g/day) - Folate (μg/day) - Vitamins A (μg/day), B1 (mg/day), D (μg/day), E (mg/day) - Potassium (mg/day) - Calcium (mg/day) - Iron (g/day)	Not mentioned	- Group I: control - Group II: patients with CRC - Group III: patients with *KRAS* mutation	- Low intake of vitamin E (OR = 2.3; 95%CI, 1.2–4.6) - Low intake of vitamin D OR = 2 (95% CI, 1.1–4.2) - Low intake of vitamin B1 OR = 2.5 (95% CI, 1.2–5.1) - Low intake of vitamin A OR = 2.5 (95% CI, 1.2–5.1) - Low intake of folate OR = 2 (95% CI, 1.1–3.9) - Low intake of fiber OR = 2.7 (95% CI, 1.4–5.1) - Low intake of calcium OR = 2.3 (95% CI, 1.1–4.6) - Low intake of vitamin A OR = 2.5 (95% CI, 1.2–5.1).
Slattery et al., 2002 [42]	Case-control study	USA: Northern California, Utah, Minnesota.	- 1836 cases - 1958 controls	white, African-American Hispanic	Association between GSTM-1 and NAT2 and colon tumors	-Cruciferous vegetables (high, intermediate, low) -Red meat frequency/day (< 0.86, 0.86–3.5, > 3.5)	Age, sex.	- Group I: *KRAS* only - Group II: *KRAS* + MSI (microsatellite instability) - Group III: *KRAS* + p53 + MSI	No significant result: - Red meat OR = 0.7 (95% CI, 0.5–1.1) - Cruciferous vegetable OR = 0.7 (95% CI 0.5–1.2)
Slattery et al., 2001 [43]	Case-control study	USA: Northern California, Utah, Minnesota.	- 1428 cases - 2410 control	White African American Hispanic	Association between lifestyle factors and *KRAS* mutations in colon cancer tumors.	- caffeine (low, intermediate, and high) - Western diet and prudent diet patterns (low, intermediate, and high).	Age.	- Group I: patients with *KRAS*^*+*^ - Group II: patients with *KRAS*^*−*^ - Group III: controls.	- For Western diet pattern, low OR = 1.0, intermediate OR = 1.2 (95% CI, 0.95–1.6), and high OR = 1.5 (95% CI, 1.2–1.9) - Prudent diet pattern showed no clear association.
Slattery et al., 2000 [44]	Case-control study	USA: Northern California, Utah, Minnesota.	- 1836 cases - 1958 controls	African-American, white, Hispanic.	Associations between dietary intake and *KRAS* mutations in colon tumors.	- Dietary fat (g/1000 kcal): fat, SFA, MUFA, PUFA, cholesterol - Insulin-related factors: Carbohydrate (g/1000 kcal), Refined grains (servings/day) - DNA methylation factors: folate (mg/1000 kcal), vitamin B6 (mg/1000 kcal), methionine (g/1000 kcal), alcohol (g/day) - Carcinogen detoxification: cruciferous vegetables.	Age, sex, energy intake, BMI, physical activity, dietary calcium, fiber.	- Group I: patients with *KRAS*^*+*^ - Group II: patients with *KRAS*^*−*^ - Group III: controls.	Low levels of vegetables OR = 0.6 (95% CI, 0.4–0.9; *P* = .01).
Kampman et al., 2000 [45]	Case control study	Netherlands	- 204 cases - 259 controls	Caucasian	Associations between animal product and *KRAS* codon 12 and 13 mutations in colon carcinomas.	- Foods: total red meat, beef, processed meat, poultry, fish, dairy products - Nutrients: total fat, SFA, cholesterol, total protein, animal protein, calcium.	Age, sex, total energy intake	- Group I: patients with *KRAS*^+^ - Group II: patients with *KRAS*^−^ - Group III: controls.	- Animal protein OR = 1.5 (95% CI, 1–2.1) for codon 12 but OR = 0.4 (95% CI, 0.2–1) for codon 13 - Calcium OR = 1.2 (95% CI, 0.9–1.6) for codon 12 but OR = 0.6 (95% CI, 0.3–1.2) for codon 13.
O’Brien et al., 2000 [46]	Case series	Norwich, United Kingdom.	51 participants (26 males and 26 females).	Unspecified	Associations between *KRAS* mutations and meat consumption in patients with left-sided CRC	Red meat (g/day)		- Group I: *KRAS*^+^ - Group II: *KRAS*^*−*^	No correlation between *KRAS* mutations and red meat consumption
Martinez et al., 1999 [47]	Case series	USA: Phoenix metropolitan area, Arizona.	678 participants	96% were white.	Associations between variables known or suspected to be related to risk of CRC and occurrence of *KRAS* mutations in colorectal adenomas.	-Total fat, SFA, dietary fiber, red meat, alcohol (g/day) - Dietary calcium, total calcium, dietary folate, total folate (mg/day).	Age, sex, energy intake.	- Group I: *KRAS*^+^ - Group II: *KRAS*^*−*^	- Only intake of total folate was associated with *KRAS* mutation; compared with individuals in the lower tertile, those in the upper tertile had 50% lower risk of having *KRAS* mutation (OR = 0.52; 95% CI, 0.30–0.88; *P* = 0.02).
Bautista et al., 1997 [48]	Case control study	Spain, Island of Majorca.	- 286 cases and 295 controls. - 106 CRC cases were available for the molecular analysis.	Unspecified	Possible associations between dietary factors and KRAS mutation in CRC tumors	- Total fats, PUFA, MUFA, SFA - calcium	Age, physical activity, number of meals, total caloric intake; fats were also adjusted for calcium, and calcium was adjusted for MUFA	- Group I: KRAS+ - Group II: KRAS- - Group III: controls	-High calcium intake was associated with a decreased risk of KRAS-mutated tumors (OR = 0.36; 95% CI, 0.14–0.97) - No association between KRAS+ and other nutrients

*Abbreviations: ANOVA* analysis of variance, *APC* adenomatous polyposis coli gene, *BMI* body mass index, *CI* confidence interval, *CRC* colorectal cancer, *GST* glutathione S-transferase, *GSTM-1* Glutathione S-transferases mu form, *HPFS* Health Professionals Follow-Up Study, *HR* hazard ratio, *MUFA* monounsaturated fatty acids, *MSI* microsatellite instability, *NAT* N-acetyltransferase, *NAT2* N-acetyltransferase 2, *NHS* Nurses’ Health Studies, *NLCS* Netherlands Cohort Study on diet and cancer, *OR* odds ratio, *PUFA* polyunsaturated fatty acids, *RR* risk ratio, *SFA* saturated fatty acids, *USA* United States of America

### Associations between foods and *KRAS* mutational status

#### Acrylamide foods

Acrylamide, which is present in heat-treated carbohydrate-rich foods such as coffee, fried/baked potatoes, and bakery goods, has been classified by the International Agency for Research on Cancer as a probable human carcinogen (group 2A) [49]. One study of the 28 included in our analysis focused on the link between *KRAS* mutations and acrylamide. This 7.3-year follow-up case-cohort analysis of 120,852 participants (58,279 men, and 62,573 women), and 733 CRC cases which were available for the molecular analysis, within the Netherlands Cohort Study on diet and cancer, reported that acrylamide intake was positively associated with risk of CRC, with activating *KRAS* mutations among men but not among women [25].

#### Alcoholic beverages

On the basis of a Case cohort study embedded in the Netherlands Cohort Study on diet and cancer (NLCS), Bongaerts et al., concluded that alcohol intake did not affect KRAS mutation status [35] but they reported a positive association with beer drinking. However, Stattery’s study shows a positive association between high level of alcohol and tumors harboring *KRAS* mutations [44]. Finally, Two case series studies found no association [31, 47].

#### Coffee and tea

The caffeine and theophylline found in coffee and tea respectively, have been shown to have no influence on the risk of colon cancer [50]. Studies on coffee and tea and their relation to *KRAS* mutations are scarce. We identified only two studies on tea [28, 36] and two studies on coffee or caffeine [28, 43], which reported no association between tea or coffee consumption and *KRAS* activating mutations.

#### Dairy products

Studies on associations between *KRAS* mutations and dairy products were inconsistent. Slattery et al. and Wark et al. found no association between dairy products and *KRAS mutation* [32, 36]*,* whereas, Kampman et al. observed that diets low in dairy products were more likely to be associated with tumors harboring *KRAS*^*+*^ mutations in codon 12 [45].

#### Fiber

We identified two case control studies which found that the high consumption of fiber was associated with reduced risk of CRC with *KRAS* mutation [32, 41].

However, a null association was reported in three studies (one was a cohort study, and two were a case series studies) [21, 31, 47].

#### Fruits and vegetables

In a case control study that described associations between vegetables and *KRAS* mutations, high-level intake of vegetables was significantly associated with reduced risk of *KRAS* mutations [32]. Two other studies included in the present systematic review showed that distribution of specific *KRAS* mutations may vary according to consumption of fruits and vegetables. In Kamal et al., patients who developed an adenoma harboring a *KRAS* codon 13 mutation consumed less fruits and vegetables and patients with *KRAS* codon 12 transversion mutations consumed more fruits and green leafy vegetables than patients with *KRAS* codon 12 transition mutations [28]. In Naguib et al., individuals harboring *KRAS*-mutated cancers with G-to-A transitions consumed less fruits and vegetables [31].

In another case-control study, low-intake levels of cruciferous vegetables were associated with reduced risk of having KRAS mutations [44]. However, Slattery’s study, showed no significant association between *KRAS* status and cruciferous vegetables intake [42].

### Meat (red meat, white meat, and fish)

#### Fish

None of the four identified studies which included fish showed an association between fish consumption and *KRAS* mutation status [31, 32, 37, 45].

#### Red meat

Identified studies had inconsistent findings regarding red meat in relation to *KRAS* mutations. Slattery et al. found in a case control study no significant association between fresh meat products and colon or rectal cancer, neither overall nor regard to *KRAS* mutation status [32, 42]. Red meat was also not associated with *KRAS* mutations in a case control study from kampman et al. [45] and case series studies from O’Brien et al. [46], and Martinez et al. [47]. However, Carr et al. reported in a case-control study with colon cancer patients, the existence of positive associations between higher red meat intake and *KRAS*^*+*^ mutations [24].

#### White meat

Naguib et al., found an association between mutations in *KRAS+* and white meat consumption [31], while Kampman et al. observed substantial differences according to the affected *KRAS* codon. They found that poultry consumption (per 17 g) was inversely associated with *KRAS* codon 13 mutation and positively association with *KRAS* codon 12 mutation [45].

#### Organochlorine compounds

Diet is an important source of exposure to many synthetic organic chemicals used in industry and agriculture, including industrial organochlorine compounds, which have been classified as “probably” or “possibly” carcinogenic to humans [49]. In a case-control study conducted in Spain, researchers found that a higher serum concentration of organochlorine compounds was associated with an elevated risk of colorectal cancer with *KRAS*^−^ but not with *KRAS*^*+*^ [40].

### Associations between nutrients and *KRAS* mutational status

#### Animal protein

High levels of animal protein have been shown to be associated with increased risk of rectal tumors [32]. A possible association with *KRAS* codon 12 mutation was highlighted in a case report study that reported the autopsy of an Italian King [30]. An association was also detected in a case-control study in which high intake of animal protein (per 17 g) was positively associated with colon tumors harboring codon 12 mutations [45].

#### Calcium

Epidemiological studies have provided mixed results regarding calcium intake and *KRAS* mutations. In fact, some researchers have reported a protective role of calcium intake, which was associated with decreased odds of having *KRAS*^*+*^ tumors [41, 48]. Other studies did not find an association between dietary calcium that was specific to tumors with *KRAS* mutations [22, 31, 36, 47]. In Kampman et al., colon tumors with codon 12 and 13 *KRAS* mutations were differently associated with intake of calcium, with positive association between calcium and mutations in codon 12) and inverse associations between calcium intake and codon 13 [45].

#### Heme iron

Heme iron is an element found exclusively in animal products and especially in red meat. Gilsing et al. showed that heme iron intake was associated with an increased risk of CRC harboring activating G > A transitions in *KRAS* mutations [27]. Laso et al., however, provided contradictory results: patients with *KRAS* mutations in codon 12 consumed significantly less iron than controls. Furthermore, a multivariate analysis for heme iron intake, adjusted by age and energy, and compared with controls and versus each molecular subtype of CRC showed no significant OR [41].

#### Fat

Fat has received much research attention for its potential impact in CRC; nonetheless, the link between fat intake and the *KRAS* mutational status in CRC is largely inconsistent. We identified a number of studies, and many did not observe an association between a high intake of total fat and risk of CRC or *KRAS* mutation status [31, 32, 44, 45, 47, 48]. Other identified studies revealed that high intake of polyunsaturated fatty acid, specifically linoleic acid, was associated with increased risk of KRAS^+^ [34, 36, 39]. Slattery et al. observed saturated and monounsaturated fats, but not polyunsaturated fat, to be associated with increased risk of colon tumors, with specific KRAS mutations at codon 12 [44]. High intake of monounsaturated fats, mostly derived from olive oil in the Spanish diet, was found to be significantly associated with decreased risk of cancer with *KRAS*^*−*^ genotype [34].

#### Vitamin a

In the present systematic review, one study showed an association between vitamin A and *KRAS* mutations in codon 12. Patients with these mutations consumed significantly less vitamin A than controls [41].

### Vitamin B (B1, B2, B6, B9, B12)

Low intake of vitamin B1 was associated with *KRAS* mutations in codon 12 but not in codon 13 in Laso et al., study [41]. Vitamin B2 was differently associated with risk of KRAS^+^ and KRAS^−^adenomas [36]. Intake of vitamin B2 was somewhat positively, but not significantly, associated with KRAS^+^ adenomas. However, Naguib’s study did not detect any association [31].

In the prospective, population-based Iowa Women’s Health Study, which included 41,836 older women, no association was observed between vitamin B6 intake and *KRAS* mutations [29]. Similarly, two another studies reported the same result [31, 44].

Findings on dietary folate or red blood cell (RBC) folate in relation to *KRAS* mutations in colorectal tumors were inconsistent in the studies included in the present systematic review. In some studies, *KRAS* mutations were not significantly associated with lack of folate [29, 31, 33, 44]. In other studies, a higher risk of *KRAS* mutations was associated with a lower intake of total folate or RBC folate [28, 41, 47]. In the study from Brink et al., differences in associations between colon and rectal cancer were observed. Dietary folate intake was not significantly associated with *KRAS* mutation status in colon cancer, but it was associated with *KRAS*-mutated tumors in rectal cancer, and the effects of folate on rectal cancer risk showed differences in men versus women [37].

Results regarding vitamin B12 were also conflicting. In the prospective, population-based Iowa Women’s Health study [29]. And in Naguib’s study researchers did not observe an association between vitamin B12 intake and overall risk of CRC or *KRAS* mutation status among older women [31]. In a study that included database information from two independent prospective cohort studies (88,691 women and 47,371 men), high vitamin B12 intake was inversely associated with colon cancer, regardless of KRAS status [33]. Conversely, low levels of vitamin B12 intake were associated with reduced risk or *KRAS* mutations in a multicenter, case-control study of colon cancer [44].

#### Vitamin C

Only one study was conducted to look for a possible association between Vitamin C intake and tumors with *KRAS* mutations, but the results found do not underline any association [31].

#### Vitamin D

Despite accumulating evidence for the preventive effect of vitamin D on colorectal carcinogenesis, its precise mechanisms remain unclear [26]. Jung et al. found that a higher predicted vitamin D score was significantly associated with lower risk of colorectal cancer, but no direct relationship with the *KRAS* gene was identified [26]. Naguib et al. reached the same result [31]. However, Laso et al. observed that *KRAS* mutations in codon 12 were significantly associated with lack of vitamin D, suggesting the protective role of this vitamin [41].

#### Vitamin E

A lower intake of vitamin E was associated with increased risk of CRC. Nonetheless, no association with *KRAS* mutations status was observed [41].

## Discussion

Acrylamide foods**:** Epidemiologic data on the effects of dietary acrylamide remain scant, with no direct evidence that dietary intake of acrylamide is associated with risk of CRC [51–53]. However, experimental studies on rodents concluded that acrylamide is carcinogenic and led to several tumors. In vivo, acrylamide is oxidized to the epoxide glycidamide that forms adducts with DNA bases and causes mutations [54]. In addition, acrylamide and glycidamide exposure have been shown to influence hormone levels in human colorectal cells by increasing the expression of genes involved in the generation of sex hormones and by affecting the ability of tumors to escape apoptosis-based surveillance mechanisms [55]. These experimental studies are concordant with Hogervorst results only in men [25].

The fact that *KRAS* mutations in women are not affected by exposure to acrylamide remains a mystery to be elucidated by experiments that take into account tumor and environmental heterogeneity, especially hormonal differences between the two sexes.

Alcoholic beverages: In a pooled analysis of eight prospective cohort studies, CRC risk was increased when daily alcohol consumption levels exceed 30.0 g [56]. This suggests that a dose-response relationship exists between alcohol consumption and CRC, with higher levels of daily alcohol intake perhaps resulting in genetic mutations [57]. In fact, mechanisms linked to colorectal tumorigenesis, such as cellular damage associated with ethanol and its metabolites, specifically acetaldehyde which can break and damage DNA leading to permanent mutations in DNA sequences; and induce reactive oxygen species formation through cytochrome pathway, have been observed in heavy or chronic alcohol consumers [58–62]. This means that studies in heavy consumers can clarify the association between alcoholic beverages and *KRAS* mutations in CRC. For moderate alcohol consumption levels, as in the studies analyzed in this systematic review, there is a certain risk of developing colorectal tumors but possibly through mechanisms other than those causative of *KRAS* mutations.

Dairy products: In a systematic review summarizing studies conducted in Middle Eastern and North African countries, some included studies reporting that dairy products were a protective factor for CRC and others considering them as a risk factor [63]. The conflicting epidemiological evidence related to dairy products might be explained by the potential dual nature of these foods. In fact, dairy products naturally contain calcium which may play a role in preventing carcinogenesis [64]. In fact, calcium is considered as a potential chemopreventive agent which reduce colon cancer by binding to some substances (acids bile and free fatty acids) whose effect is toxic on colonic epithelial cells; and by inducing the cell differentiation when it enters the interior of these cells [65]. On the other hand, dairy products may also contain fats, hormones and growth factors, which can promote tumor growth [65, 66], and may explain the results inconsistency found in this systematic review.

Fiber: A review of all published meta-analyses was performed on the association between dietary fiber and colorectal cancer conclude that the high consumption of fiber may benefit from a reduction in the incidence of developing colorectal cancer [67]. High-fiber content has many roles, including diluting and binding potential carcinogens as well as reducing transit time [68, 69]. Therefore, it was not surprising to find that dietary fiber was significantly associated with reduced risk of rectal tumors overall as well as with reduced risk of *KRAS* tumor mutations [32, 41].

Fruits and vegetables: Fruits and vegetables contain many nutrients and phytochemicals that have antioxidant, antimutagenic, and anticarcinogenic properties [70, 71]. The relation between fruit and vegetable consumption and the low risk of *KRAS* mutational status may be due to their richness in fiber and some bioactive compounds such as flavonols, which are capable of inhibiting nitroso compound formation [72]. Nitroso compounds are capable of inducing guanine base alkylation, which, if not repaired, can lead to G to A base transitions [73].

Fish: although it has been long believed that n-3 fatty acids (more popularly known as omega-3 fatty acids), which are present in high levels in fish, are capable of preventing carcinogenesis via multiple pathways [74, 75], increasing studies have shown conflicting findings between omega-3 fatty acids and cancer prevention. In fact, a reanalysis of past research has suggested that there may not be any reduction of cancer risk after all [76], or the association between fish and CRC is very weak and differs according to gender and countries with no non-linear dose-response association [77]. Genetic Studies in this review confirm previous findings, showing no mutagenic or protective effect of fish.

Red meat: studies have shown that red meat is strongly associated with increasing CRC risk by approximately 20% with increasing intake of dairy up to ~ 150 g/d [78, 79]. Barbequed red meat or meat prepared at high temperatures may be important sources of mutagenic and carcinogenic compounds, such as heterocyclic amines [80], and tumor promoters [81]. Red meat consumption may increase colon cancer risk by inducing the endogenous production of N-nitroso compounds and their precursors, which may induce *KRAS* mutations [82, 83].

In the prospective cohort study from Brink et al., the absence of an association between fresh meat and risk of colon or rectal cancer with *KRAS* mutations could be due to the expected low content of carcinogens in the fresh meat preparations consumed by the participants and also to the lack of correlations between meat preparation and the amount of fresh meat consumed [37].

White meat: epidemiological studies generally show that there is no association between white meat consumption and CRC risk [84]. Kampman et al. suggest that colon tumors with codon 12 and 13 *KRAS* mutations are related differently to consumption of poultry [45]. In vitro mechanistic investigations are needed to elucidate these contradictory findings.

Organochlorine compounds: only one study treated this topic with a small sample [40]. There results remain hypothesis generating and further studies are needed to address the questions about the association between *KRAS* mutations and organochlorine compounds.

Animal protein: In animal and in vitro studies, a high protein diet could lead to DNA damage of colonocytes and decrease colonic mucosal thickness [85], but epidemiological data remain controversial. Two studies in this review agree on a possible positive association between animal protein and *KRAS*^*+*^ mutation [32, 45]. However, a meta-analysis comprising 8187 cases, concluded that there was no relationship between animal or vegetable protein and CRC risk [85].

Calcium: the variations in reports of calcium intake being linked to CRC may be partly due to alterations in bile acids, which are carcinogenic in animal models. In fact, studies in animals have indicated a protective effect of dietary calcium, which binds bile acids in the bowel lumen, inhibiting their proliferative and the carcinogenic effects [86, 87], and reduces the number of mutations in the *KRAS* gene of the rat [88]. Also, a recent meta-analysis including 14 cohort studies and 15 case-control studies, suggests that higher intakes of dietary Calcium may help to reduce the risk of CRC slightly [89].

Heme iron: in a meta-analysis of five prospective studies, the relative risk of colon cancer was 1.18 (95% CI, 1.06–1.32) for patients who had the highest category of heme iron intake compared with those in the lowest category [90]. Heme iron and its metabolic products may increase the overall mutation rate and promote specific point mutations in the DNA of colonic tissue. For example, heme was shown to catalyze the endogenous formation of *N-*nitroso compounds [91, 92]. Such metabolites have been shown to induce G > A transitions in a variety of genes, including *KRAS,* in both rodent and in vitro studies [93].

Fat: The association between total dietary fat, including fat constituents such as saturated fat, monounsaturated fat, polyunsaturated fat, and cholesterol, and CRC risk has been evaluated in numerous epidemiologic studies. Results have generally been mixed [94]. In our review, we note the same finding regarding *KRAS* mutations: whereas some studies have reported positive associations, several studies have observed null or inverse associations. However, some recent studies underline the fat carcinogenic potential [95]. The main hypothesis supporting a possible effect of fat on CRC risk is based on the intraluminal effect of the fat digestion products. In fact, fat may promote colon cellular damage by increasing bile acid and fatty acid excretion in the colonic lumen [96].

Vitamins: B vitamins are essential for DNA methylation and nucleotide biosynthesis. Adequate dietary intake of these vitamins has previously been related to a lower colon cancer risk [97]. Folic acid, a water-soluble B-complex vitamin (B9) is the most studied and the most controversial. In fact, the relationship between folate intake or blood levels and colorectal molecular parameters appears to be quite complex. The studies included in the present systematic review showed inconsistent results that may differ depending on gender, tumor position, folic acid concentration and type of *KRAS* mutations. There is evidence for a “dual role” of folate in carcinogenesis whereby folate may prevent early cancers but causes harm if the lesions have formed [98]. Despite this effect, epidemiological studies have shown that reduced folate levels may be a risk factor for *KRAS* mutation development, especially because folate is an important coenzyme for DNA methylation and synthesis. However, a meta-analysis of 27 papers showed a relative risk estimate reduction of 0.85 (95% CI, 0.74–0.99) when comparing low versus high folic acid supplementation [99]. The absence of consistent epidemiological evidence regarding folic acid intake or blood levels and *KRAS* mutational status suggests that more work is still needed to fully delineate the influence of this nutrient on CRC molecular subtypes.

Vitamin D: the consistent and significant inverse association between vitamin D and risk of CRC was supported by experimental studies, which demonstrated that vitamin D reduces proliferation, inflammation, and angiogenesis, stimulates differentiation and apoptosis, and enhances the immune system [100, 101]. A recent meta-analysis including 15 studies in 14 countries has achieved an important result: having a 25(OH) D concentration > 35 ng/ml was associated with a nearly 40% lower risk of colorectal cancer compared with < 15 ng/ml [102].

In summary, the present systematic review of epidemiological studies focusing in the associations between nutritional factors and *KRAS* mutations in CRC found that there is no association between fish, vitamin C, coffee and tea consumption, and *KRAS* mutation status in CRC. High levels of animal protein, acrylamide foods, and low levels of vitamin A consumption have been shown to be associated with increased risk of CRC tumors with *KRAS* mutations. However, concerning alcoholic beverages, dairy products, fiber, fruits and vegetables, red meat, calcium, heme iron, fat and vitamin B inconsistent and conflicting results have been found between these nutritional factors and specific *KRAS* mutations in CRC.

These inconsistencies may be explained by the strength of the evidence, which varied widely depending on study design. Although case-control studies were more likely to find a significant association between a nutritional factor of interest and *KRAS* mutations, these significant associations did not carry over to cohort studies, which are considered to have a stronger and more robust design, free of the limitations that accompany case-control studies. Inconsistent findings of epidemiological studies on *KRAS* mutations in CRC and nutritional factors may have been also due to small sample sizes in several studies, measurement error, and confounding variables. In addition, some studies conclude that codon 12 has a preferential association with codon 13, which shows that in the same gene, the susceptibility to mutations through nutrients can vary, making the gene-nutrition association very complex. Along with these considerations, associations between diet and *KRAS* mutational status may be hard to confirm in epidemiological studies that used questionnaires to assess exposure. Such assessments may not be sensitive enough to detect associations; biological assessment of specific nutrients hypothesized to affect KRAS mutations in body fluids or tissue samples may be more appropriate.

## Conclusion

Some epidemiological studies on diet and colorectal *KRAS* mutations are highly inconsistent and conflicting; others were homogeneous especially for fish, vitamins C and E, coffee, tea, animal protein, acrylamide foods, and vitamin A. Further studies on epidemiological associations with a more robust prospective cohort design are needed. In addition, there is a need for investigations on the most effective way to implement what is already known about healthy nutrition choices, thereby allowing risk of CRC and other cancers related to diet to be decreased.

## Data Availability

All data generated or analysed during this study are included in this published article.

## Abbreviations

ANOVA: Analysis of variance
APC: Adenomatous polyposis coli gene
BMI: Body mass index
CI: Confidence interval
CRC: Colorectal cancer
GST: Glutathione S-transferase
GSTM-1: Glutathione S-transferases mu form
HPFS: Health professionals follow-up study
HR: Hazard ratio
KRAS: Kristen rat sarcoma
*KRAS -*: Wild-type *KRAS*
*KRAS* +: Mutant *KRAS*
MUFA: Monounsaturated fatty acids
MSI: Microsatellite instability
NAT: N-acetyltransferase
NAT2: N-acetyltransferase 2
NHS: Nurses’ health studies
NLCS: Netherlands cohort study on diet and cancer
OR: Odds ratio
P53: Tumor protein 53
PRISMA: Preferred reporting items for systematic reviews and meta-analyses guidelines
PUFA: Polyunsaturated fatty acids
RR: Risk ratio
SFA: Saturated fatty acids

## References

[1] Bray F, Ferlay J, Soerjomataram I, Siegel RL, Torre LA, Jemal A (2018). Global cancer statistics 2018: GLOBOCAN estimates of incidence and mortality worldwide for 36 cancers in 185 countries. CA Cancer J Clin.

[2] Lao VV, Grady WM (2011). Epigenetics and colorectal cancer. Nat Rev Gastroenterol Hepatol.

[3] Markowitz SD, Bertagnolli MM (2009). Molecular origins of cancer: Molecular basis of colorectal cancer. N Engl J Med.

[4] Beggs AD, Hodgson SV (2008). The genomics of colorectal Cancer: state of the art. Curr Genomics.

[5] Lièvre A, Bachet J-B, Le Corre D, Boige V, Landi B, Emile J-F (2006). KRAS mutation status is predictive of response to cetuximab therapy in colorectal cancer. Cancer Res.

[6] Peeters M, Kafatos G, Taylor A, Gastanaga VM, Oliner KS, Hechmati G (2015). Prevalence of RAS mutations and individual variation patterns among patients with metastatic colorectal cancer: A pooled analysis of randomised controlled trials. Eur J Cancer Oxf Engl 1990.

[7] Kudryavtseva AV, Lipatova AV, Zaretsky AR, Moskalev AA, Fedorova MS, Rasskazova AS (2016). Important molecular genetic markers of colorectal cancer. Oncotarget.

[8] Phipps AI, Buchanan DD, Makar KW, Win AK, Baron JA, Lindor NM (2013). KRAS-mutation status in relation to colorectal cancer survival: the joint impact of correlated tumour markers. Br J Cancer.

[9] Benvenuti S, Sartore-Bianchi A, Di Nicolantonio F, Zanon C, Moroni M, Veronese S (2007). Oncogenic activation of the RAS/RAF signaling pathway impairs the response of metastatic colorectal cancers to anti-epidermal growth factor receptor antibody therapies. Cancer Res.

[10] Grady WM, Pritchard CC (2014). Molecular alterations and biomarkers in colorectal cancer. Toxicol Pathol.

[11] Heinemann V, Stintzing S, Kirchner T, Boeck S, Jung A (2009). Clinical relevance of EGFR- and KRAS-status in colorectal cancer patients treated with monoclonal antibodies directed against the EGFR. Cancer Treat Rev.

[12] Saif MW, Shah M (2009). K-ras mutations in colorectal cancer: a practice changing discovery. Clin Adv Hematol Oncol.

[13] Bazan V, Agnese V, Corsale S, Calò V, Valerio MR, Latteri MA (2005). Specific TP53 and/or Ki-ras mutations as independent predictors of clinical outcome in sporadic colorectal adenocarcinomas: results of a 5-year Gruppo Oncologico dell’Italia Meridionale (GOIM) prospective study. Ann Oncol Off J Eur Soc Med Oncol.

[14] Hou TY, Davidson LA, Kim E, Fan Y-Y, Fuentes NR, Triff K (2016). Nutrient-Gene Interaction in Colon Cancer, from the Membrane to Cellular Physiology. Annu Rev Nutr.

[15] Ramzi NH, Chahil JK, Lye SH, Munretnam K, Sahadevappa KI, Velapasamy S (2014). Role of genetic & environment risk factors in the aetiology of colorectal cancer in Malaysia. Indian J Med Res.

[16] Ashkavand Z, O’Flanagan C, Hennig M, Du X, Hursting SD, Krupenko SA (2017). Metabolic reprogramming by folate restriction leads to a less aggressive cancer phenotype. Mol Cancer Res.

[17] Davidson LA, Lupton JR, Jiang YH, Chapkin RS (1999). Carcinogen and dietary lipid regulate ras expression and localization in rat colon without affecting farnesylation kinetics. Carcinogenesis.

[18] Hu Y, McIntosh GH, Le Leu RK, Woodman R, Young GP (2008). Suppression of colorectal oncogenesis by selenium-enriched milk proteins: apoptosis and K-ras mutations. Cancer Res.

[19] Moher D, Liberati A, Tetzlaff J, Altman DG (2009). PRISMA Group. Preferred reporting items for systematic reviews and meta-analyses: the PRISMA statement. Ann Intern Med.

[20] Sackett DL (1989). Rules of evidence and clinical recommendations on the use of antithrombotic agents. Chest.

[21] He X, Wu K, Zhang X, Nishihara R, Cao Y, Fuchs CS (2019). Dietary intake of fiber, whole grains and risk of colorectal cancer: An updated analysis according to food sources, tumor location and molecular subtypes in two large US cohorts. Int J Cancer.

[22] Keum N, Liu L, Hamada T, Qian ZR, Nowak JA, Cao Y (2019). Calcium intake and colon cancer risk subtypes by tumor molecular characteristics. Cancer Causes Control.

[23] Mehta RS, Song M, Nishihara R, Drew DA, Wu K, Qian ZR (2017). Dietary Patterns and Risk of Colorectal Cancer: Analysis by Tumor Location and Molecular Subtypes. Gastroenterology.

[24] Carr PR, Jansen L, Bienert S, Roth W, Herpel E, Kloor M (2017). Associations of red and processed meat intake with major molecular pathological features of colorectal cancer. Eur J Epidemiol.

[25] Hogervorst JGF, de Bruijn-Geraets D, Schouten LJ, van Engeland M, de Kok TMCM, Goldbohm RA (2014). Dietary acrylamide intake and the risk of colorectal cancer with specific mutations in KRAS and APC. Carcinogenesis.

[26] Jung S, Qian ZR, Yamauchi M, Bertrand KA, Fitzgerald KC, Inamura K (2014). Predicted 25(OH) D score and colorectal cancer risk according to vitamin D receptor expression. Cancer Epidemiol Biomark Prev.

[27] Gilsing AMJ, Fransen F, de Kok TM, Goldbohm AR, Schouten LJ, de Bruïne AP (2013). Dietary heme iron and the risk of colorectal cancer with specific mutations in KRAS and APC. Carcinogenesis.

[28] Kamal MM, Youssef OZ, Lotfy AN, Elsaed ET, Fawzy MMT (2012). Association of folate intake, dietary habits, smoking and COX-2 promotor -765G>C polymorphism with K-ras mutation in patients with colorectal cancer. J Egypt Natl Cancer Inst.

[29] Razzak AA, Oxentenko AS, Vierkant RA, Tillmans LS, Wang AH, Weisenberger DJ (2012). Associations between intake of folate and related micronutrients with molecularly defined colorectal cancer risks in the Iowa Women’s health study. Nutr Cancer.

[30] Ottini L, Falchetti M, Marinozzi S, Angeletti LR, Fornaciari G (2011). Gene-environment interactions in the pre-industrial era: the cancer of king Ferrante I of Aragon (1431-1494). Hum Pathol.

[31] Naguib A, Mitrou PN, Gay LJ, Cooke JC, Luben RN, Ball RY (2010). Dietary, lifestyle and clinicopathological factors associated with BRAF and K-ras mutations arising in distinct subsets of colorectal cancers in the EPIC Norfolk study. BMC Cancer.

[32] Slattery ML, Curtin K, Wolff RK, Herrick JS, Caan BJ, Samowitz W (2010). Diet, physical activity, and body size associations with rectal tumor mutations and epigenetic changes. Cancer Causes Control.

[33] Schernhammer ES, Giovannuccci E, Fuchs CS, Ogino S (2008). A prospective study of dietary folate and vitamin B and colon cancer according to microsatellite instability and KRAS mutational status. Cancer Epidemiol Biomark Prev.

[34] Weijenberg MP, Lüchtenborg M, de Goeij AFPM, Brink M, van Muijen GNP, de Bruïne AP (2007). Dietary fat and risk of colon and rectal cancer with aberrant MLH1 expression, APC or KRAS genes. Cancer Causes Control.

[35] Bongaerts BWC, de Goeij AFPM, van den Brandt PA, Weijenberg MP (2006). Alcohol and the risk of colon and rectal cancer with mutations in the K-ras gene. Alcohol.

[36] Wark PA, Van der Kuil W, Ploemacher J, Van Muijen GNP, Mulder CJJ, Weijenberg MP (2006). Diet, lifestyle and risk of K-ras mutation-positive and -negative colorectal adenomas. Int J Cancer.

[37] Brink M, Weijenberg MP, de Goeij AFPM, Roemen GMJM, Lentjes MHFM, de Bruïne AP (2005). Meat consumption and K-ras mutations in sporadic colon and rectal cancer in The Netherlands Cohort Study. Br J Cancer.

[38] Brink M, Weijenberg MP, de Goeij AFPM, Roemen GMJM, Lentjes MHFM, de Bruïne AP (2005). Dietary folate intake and k-ras mutations in sporadic colon and rectal cancer in The Netherlands Cohort Study. Int J Cancer.

[39] Brink M, Weijenberg MP, De Goeij AFPM, Schouten LJ, Koedijk FDH, Roemen GMJM (2004). Fat and K-ras mutations in sporadic colorectal cancer in the Netherlands cohort study. Carcinogenesis.

[40] Howsam M, Grimalt JO, Guinó E, Navarro M, Martí-Ragué J, Peinado MA (2004). Organochlorine exposure and colorectal cancer risk. Environ Health Perspect.

[41] Laso N, Mas S, Jose Lafuente M, Casterad X, Trias M, Ballesta A (2004). Decrease in specific micronutrient intake in colorectal cancer patients with tumors presenting Ki-ras mutation. Anticancer Res.

[42] Slattery ML, Curtin K, Ma K, Schaffer D, Potter J, Samowitz W (2002). GSTM-1 and NAT2 and genetic alterations in colon tumors. Cancer Causes Control.

[43] Slattery ML, Anderson K, Curtin K, Ma K, Schaffer D, Edwards S (2001). Lifestyle factors and Ki-ras mutations in colon cancer tumors. Mutat Res.

[44] Slattery ML, Curtin K, Anderson K, Ma KN, Edwards S, Leppert M (2000). Associations between dietary intake and Ki-ras mutations in colon tumors: a population-based study. Cancer Res.

[45] Kampman E, Voskuil DW, van Kraats AA, Balder HF, van Muijen GN, Goldbohm RA (2000). Animal products and K-ras codon 12 and 13 mutations in colon carcinomas. Carcinogenesis.

[46] O’Brien H, Matthew JA, Gee JM, Watson M, Rhodes M, Speakman CT (2000). K-ras mutations, rectal crypt cells proliferation, and meat consumption in patients with left-sided colorectal carcinoma. Eur J Cancer Prev.

[47] Martínez ME, Maltzman T, Marshall JR, Einspahr J, Reid ME, Sampliner R (1999). Risk factors for Ki-ras protooncogene mutation in sporadic colorectal adenomas. Cancer Res.

[48] Bautista D, Obrador A, Moreno V, Cabeza E, Canet R, Benito E (1997). Ki-ras mutation modifies the protective effect of dietary monounsaturated fat and calcium on sporadic colorectal cancer. Cancer Epidemiol Biomark Prev.

[49] IARC. Occupational exposures in insecticide application, and some pesticides. In 1991. p. 179–250. (IARC Monogr Eval Carcinog Risk Hum). PMC7682260 1688189

[50] Vieira AR, Abar L, Chan D, Vingeliene S, Polemiti E, Stevens C, et al. Foods and beverages and colorectal cancer risk: a systematic review and meta-analysis of cohort studies, an update of the evidence of the WCRF-AICR Continuous Update Project. Ann Oncol. 2017;28(8):1788–802. 10.1093/annonc/mdx171. 10.1093/annonc/mdx171 28407090

[51] Larsson SC, Akesson A, Bergkvist L, Wolk A (2009). Dietary acrylamide intake and risk of colorectal cancer in a prospective cohort of men. Eur J Cancer Oxf Engl 1990.

[52] Mucci LA, Adami H-O, Wolk A (2006). Prospective study of dietary acrylamide and risk of colorectal cancer among women. Int J Cancer.

[53] Pelucchi C, Galeone C, Levi F, Negri E, Franceschi S, Talamini R (2006). Dietary acrylamide and human cancer. Int J Cancer.

[54] Besaratinia A, Pfeifer GP, Acrylamide B (2004). Genotoxicity of acrylamide and glycidamide.

[55] Clement FC, Dip R, Naegeli H (2007). Expression profile of human cells in culture exposed to glycidamide, a reactive metabolite of the heat-induced food carcinogen acrylamide. Toxicology.

[56] Cho E, Smith-Warner SA, Ritz J, van den Brandt PA, Colditz GA, Folsom AR (2004). Alcohol intake and colorectal cancer: a pooled analysis of 8 cohort studies. Ann Intern Med.

[57] Wang Y, Duan H, Yang H, Lin J (2015). A pooled analysis of alcohol intake and colorectal cancer. Int J Clin Exp Med.

[58] Badger TM, Ronis MJJ, Seitz HK, Albano E, Ingelman-Sundberg M, Lieber CS (2003). Alcohol metabolism: role in toxicity and carcinogenesis. Alcohol Clin Exp Res.

[59] Boffetta P, Hashibe M (2006). Alcohol and cancer. Lancet Oncol.

[60] Seitz HK, Maurer B, Stickel F (2005). Alcohol consumption and cancer of the gastrointestinal tract. Dig Dis Basel Switz..

[61] Taylor B, Rehm J (2005). Moderate alcohol consumption and diseases of the gastrointestinal system: a review of pathophysiological processes. Dig Dis Basel Switz.

[62] Garaycoechea JI, Crossan GP, Langevin F, Mulderrig L, Louzada S, Yang F (2018). Alcohol and endogenous aldehydes damage chromosomes and mutate stem cells. Nature.

[63] El Kinany K, Deoula M, Hatime Z, Bennani B, El Rhazi K (2018). Dairy products and colorectal cancer in middle eastern and north African countries: a systematic review. BMC Cancer.

[64] Ingraham BA, Bragdon B, Nohe A (2008). Molecular basis of the potential of vitamin D to prevent cancer. Curr Med Res Opin.

[65] Norat T, Riboli E (2003). Dairy products and colorectal cancer. A review of possible mechanisms and epidemiological evidence. Eur J Clin Nutr.

[66] Maruyama K, Oshima T, Ohyama K (2010). Exposure to exogenous estrogen through intake of commercial milk produced from pregnant cows. Pediatr Int.

[67] McRae MP (2018). The Benefits of Dietary Fiber Intake on Reducing the Risk of Cancer: An Umbrella Review of Meta-analyses. J Chiropr Med.

[68] Kim YI (2000). AGA technical review: impact of dietary fiber on colon cancer occurrence. Gastroenterology.

[69] O’Keefe SJD, Li JV, Lahti L, Ou J, Carbonero F, Mohammed K (2015). Fat, fibre and cancer risk in African Americans and rural Africans. Nat Commun.

[70] Lampe JW (1999). Health effects of vegetables and fruit: assessing mechanisms of action in human experimental studies. Am J Clin Nutr.

[71] Steinmetz KA, Potter JD. Vegetables, fruit, and cancer. II Mechanisms Cancer Causes Control 1991;2(6):427–442. 10.1007/BF00054304 1764568

[72] Lee SYH, Munerol B, Pollard S, Youdim KA, Pannala AS, Kuhnle GGC (2006). The reaction of flavanols with nitrous acid protects against N-nitrosamine formation and leads to the formation of nitroso derivatives which inhibit cancer cell growth. Free Radic Biol Med.

[73] Saffhill R, Margison GP, O’Connor PJ (1985). Mechanisms of carcinogenesis induced by alkylating agents. Biochim Biophys Acta.

[74] Pham NM, Mizoue T, Tanaka K, Tsuji I, Tamakoshi A, Matsuo K (2014). Meat consumption and colorectal cancer risk: an evaluation based on a systematic review of epidemiologic evidence among the Japanese population. Jpn J Clin Oncol.

[75] Cockbain AJ, Toogood GJ, Hull MA (2012). Omega-3 polyunsaturated fatty acids for the treatment and prevention of colorectal cancer. Gut.

[76] American Cancer Society (ACR) (2013). Omega-3 Fatty Acids.

[77] Schwingshackl L, Schwedhelm C, Hoffmann G, Knüppel S, Laure Preterre A, Iqbal K (2018). Food groups and risk of colorectal cancer. Int J Cancer.

[78] Smolińska K, Paluszkiewicz P (2010). Risk of colorectal cancer in relation to frequency and total amount of red meat consumption. Systematic review and meta-analysis. Arch Med Sci AMS.

[79] Aykan NF. Red meat and colorectal cancer. Oncol Rev. 2015;9(1):288. 10.4081/oncol.2015.288. 10.4081/oncol.2015.288 PMC4698595 26779313

[80] Sugimura T, Sato S (1983). Mutagens-carcinogens in foods. Cancer Res.

[81] Corpet DE, Stamp D, Medline A, Minkin S, Archer MC, Bruce WR (1990). Promotion of colonic microadenoma growth in mice and rats fed cooked sugar or cooked casein and fat. Cancer Res.

[82] Tachino N, Hayashi R, Liew C, Bailey G, Dashwood R (1995). Evidence for ras gene mutation in 2-amino-3-methylimidazo[4,5-f]quinoline-induced colonic aberrant crypts in the rat. Mol Carcinog.

[83] Ziegel R, Shallop A, Jones R, Tretyakova N (2003). K-ras gene sequence effects on the formation of 4-(methylnitrosamino)-1-(3-pyridyl)-1-butanone (NNK)-DNA adducts. Chem Res Toxicol.

[84] Lippi G, Mattiuzzi C, Cervellin G (2016). Meat consumption and cancer risk: a critical review of published meta-analyses. Crit Rev Oncol Hematol.

[85] Lai R, Bian Z, Lin H, Ren J, Zhou H, Guo H (2017). The association between dietary protein intake and colorectal cancer risk: a meta-analysis. World J Surg Oncol.

[86] Newmark HL, Wargovich MJ, Bruce WR (1984). Colon cancer and dietary fat, phosphate, and calcium: a hypothesis. J Natl Cancer Inst.

[87] Pence BC (1993). Role of calcium in colon cancer prevention: experimental and clinical studies. Mutat Res.

[88] Llor X, Jacoby RF, Teng BB, Davidson NO, Sitrin MD, Brasitus TA (1991). K-ras mutations in 1,2-dimethylhydrazine-induced colonic tumors: effects of supplemental dietary calcium and vitamin D deficiency. Cancer Res.

[89] Meng Y, Sun J, Yu J, Wang C, Su J. Dietary intakes of calcium, iron, magnesium, and potassium elements and the risk of colorectal cancer: a meta-analysis. Biol Trace Elem Res. 2018;189:325–35. 10.1007/s12011-018-1474-z 30171595

[90] Bastide NM, Pierre FHF, Corpet DE (2011). Heme iron from meat and risk of colorectal cancer: a meta-analysis and a review of the mechanisms involved. Cancer Prev Res.

[91] Cross AJ, Pollock JRA, Bingham SA (2003). Haem, not protein or inorganic iron, is responsible for endogenous intestinal N-nitrosation arising from red meat. Cancer Res.

[92] Hebels DGAJ, Briedé JJ, Khampang R, Kleinjans JCS, de Kok TMCM (2010). Radical mechanisms in nitrosamine- and nitrosamide-induced whole-genome gene expression modulations in Caco-2 cells. Toxicol Sci Off J Soc Toxicol.

[93] Jacoby RF, Alexander RJ, Raicht RF, Brasitus TA (1992). K-ras oncogene mutations in rat colon tumors induced by N-methyl-N-nitrosourea. Carcinogenesis.

[94] Alexander DD, Cushing CA, Lowe KA, Sceurman B, Roberts MA (2009). Meta-analysis of animal fat or animal protein intake and colorectal cancer. Am J Clin Nutr.

[95] O’Keefe SJD (2016). Diet, microorganisms and their metabolites, and colon cancer. Nat Rev Gastroenterol Hepatol.

[96] Van der Meer R, Kleibeuker JH, Lapré JA (1991). Calcium phosphate, bile acids and colorectal cancer. Eur J Cancer Prev.

[97] Harnack L, Jacobs DR, Nicodemus K, Lazovich D, Anderson K, Folsom AR (2002). Relationship of folate, vitamin B-6, vitamin B-12, and methionine intake to incidence of colorectal cancers. Nutr Cancer.

[98] Burr NE, Hull MA, Subramanian V (2017). Folic acid supplementation may reduce colorectal Cancer risk in patients with inflammatory bowel disease: a systematic review and meta-analysis. J Clin Gastroenterol.

[99] Kennedy DA, Stern SJ, Moretti M, Matok I, Sarkar M, Nickel C (2011). Folate intake and the risk of colorectal cancer: a systematic review and meta-analysis. Cancer Epidemiol.

[100] Krishnan AV, Feldman D (2011). Mechanisms of the anti-cancer and anti-inflammatory actions of vitamin D. Annu Rev Pharmacol Toxicol.

[101] Deeb KK, Trump DL, Johnson CS (2007). Vitamin D signalling pathways in cancer: potential for anticancer therapeutics. Nat Rev Cancer.

[102] Garland CF, Gorham ED (2017). Dose-response of serum 25-hydroxyvitamin D in association with risk of colorectal cancer: a meta-analysis. J Steroid Biochem Mol Biol.

